# Heat-Associated Mortality and Emergency Department Utilization in Southern Nevada During 2024: A Retrospective Descriptive Analysis

**DOI:** 10.7759/cureus.107647

**Published:** 2026-04-24

**Authors:** Deanna Chea, Justin B Atkins, Oscar A Toro Ruilowa, Lisa Durette

**Affiliations:** 1 Internal Medicine, University of Nevada, Las Vegas School of Medicine, Las Vegas, USA; 2 Medicine, University of Nevada, Las Vegas School of Medicine, Las Vegas, USA; 3 Psychiatry, University of Nevada, Las Vegas School of Medicine, Las Vegas, USA; 4 Psychiatry and Behavioral Health, University of Nevada, Las Vegas School of Medicine, Las Vegas, USA

**Keywords:** climate change, emergency department utilization, extreme heat, health disparities, heat-associated mortality, homelessness, nevada, substance use

## Abstract

Background

Extreme heat is the leading cause of weather-related mortality in the United States and is increasing in frequency and severity due to climate change. Southern Nevada experiences prolonged summer temperatures frequently exceeding 110°F, creating sustained environmental exposure and elevated risk for heat-related illness and death.

Methods

A retrospective descriptive analysis was conducted using publicly available data on heat-associated deaths identified by the Clark County Office of the Coroner/Medical Examiner and heat-related emergency department (ED) visits captured through syndromic surveillance. Demographic characteristics, housing status, substance involvement, temporal trends, and geographic distribution were analyzed.

Results

In 2024, Clark County recorded 513 heat-associated deaths, representing a 73% increase from 2023, and 3,548 heat-related ED visits. Mortality disproportionately affected males (n=395; 77%), middle-aged and older adults, individuals experiencing homelessness (n=174; 34%), and those with documented substance involvement (n=287; 56%), most commonly methamphetamine. Mortality and ED visits increased sharply during periods when daily temperatures exceeded 110°F, with peak deaths occurring in July. Geographic analysis demonstrated clustering of deaths in lower-income central and eastern ZIP codes, while several higher-income areas reported few or no deaths.

Conclusions

Heat-associated mortality in Southern Nevada is not evenly distributed but concentrated among populations with overlapping social and behavioral vulnerabilities. These findings suggest that structural determinants of health, including housing instability, substance use, and neighborhood-level inequities, are associated with heat-related risk. As extreme heat events become more frequent, targeted, population-specific public health interventions will be critical to reducing preventable mortality.

## Introduction

Heat exposure is a well-established environmental determinant of morbidity and mortality, with elevated ambient temperatures associated with increased all-cause mortality across diverse geographic settings. Epidemiologic studies consistently demonstrate a nonlinear relationship between temperature and mortality, with risk increasing sharply at higher temperature thresholds, particularly during sustained heat events [1-3]. Extreme heat is the leading cause of weather-related mortality in the United States and is increasing in frequency and severity, with national surveillance data demonstrating rising heat-related illness and mortality trends [4,5]. As climate change intensifies the frequency, duration, and severity of extreme heat events, heat exposure has emerged as a critical and expanding public health threat.

Beyond its direct physiologic effects, heat exposure interacts with behavioral, psychiatric, and social factors that amplify risk. Heat stress can impair thermoregulation and exacerbate underlying medical conditions, including cardiovascular and neurologic disease [6]. Emerging evidence also demonstrates that elevated temperatures are associated with worsening mental health outcomes, including increased psychiatric morbidity, substance use-related complications, and higher rates of suicide and crisis presentations [7-11]. These findings suggest that heat-related mortality is not solely a function of environmental exposure, but the product of complex interactions between physiologic vulnerability and behavioral health risk.

Substance use represents an additional and underrecognized contributor to heat-related morbidity and mortality. Stimulants such as methamphetamine can disrupt thermoregulation, increase metabolic heat production, and impair behavioral responses to heat exposure, thereby increasing the risk of hyperthermia and death [12-15]. The intersection of substance use, psychiatric illness, and environmental heat exposure may therefore represent a particularly high-risk pathway for adverse outcomes, especially in populations with limited access to care or protective resources.

Social and structural factors further shape vulnerability to heat-related illness and death. Individuals experiencing homelessness are disproportionately exposed to environmental extremes and often lack access to cooling, hydration, and medical care, placing them at markedly increased risk during heat events [16,17]. More broadly, heat-related mortality is unevenly distributed across populations, with increased risk among individuals with lower socioeconomic status, limited social support, and reduced access to infrastructure and resources [18,19]. These disparities are further reinforced by characteristics of the built environment, including urban heat island effects and historically patterned inequities in housing and neighborhood development, which result in higher heat exposure in socioeconomically disadvantaged communities [20-23].

These patterns are consistent with broader frameworks of social determinants of health and structural vulnerability, which emphasize that health outcomes are shaped not only by individual risk factors but also by access to resources that enable individuals to avoid risks or mitigate their consequences [24-26]. In the context of extreme heat, factors including housing stability, access to cooling, behavioral health resources, and community infrastructure play a central role in determining who is most at risk.

Southern Nevada represents a particularly high-risk environment for heat-related illness and mortality. The region experiences prolonged summer heat, with temperatures frequently exceeding thresholds associated with increased mortality. At the same time, Clark County has a growing population of individuals experiencing homelessness, high rates of substance use, and marked socioeconomic disparities, creating conditions in which environmental exposure and structural vulnerability intersect.

The objective of this study was to characterize heat-associated deaths and emergency department (ED) visits in Clark County, Nevada, during 2024. Specifically, we examined demographic patterns, substance involvement, temporal trends, and geographic distribution of heat-associated mortality and morbidity. By framing these findings within a public health and structural vulnerability context, this study aims to provide a clearer understanding of how environmental exposure, behavioral health factors, and social conditions intersect to shape heat-related risk and inform targeted interventions for high-risk populations.

## Materials and methods

Study design and setting

This study is a retrospective, observational analysis of heat-associated mortality and ED visits in Clark County, Nevada, during the 2024 calendar year. Clark County, which includes the Las Vegas metropolitan area, is characterized by a desert climate with prolonged periods of extreme heat during the summer months, making it a high-risk environment for heat-related illness.

Data sources

Data were obtained from the Southern Nevada Health District (SNHD), which conducts routine surveillance of heat-associated illness and mortality [27]. Mortality data were derived from SNHD surveillance conducted in collaboration with the Clark County Office of the Coroner/Medical Examiner. Heat-associated deaths were identified based on standard investigative criteria, including environmental exposure history, clinical presentation, and postmortem findings. 

ED visit data were obtained through syndromic surveillance from the National Syndromic Surveillance Program (NSSP), as reported by SNHD. Heat-related ED visits were identified using predefined syndrome definitions incorporating chief complaint text, discharge diagnoses, and clinical indicators consistent with heat-related illness. Syndromic surveillance definitions (NSSP) and classification of heat-associated deaths by the coroner/medical examiner follow standardized institutional protocols; however, detailed query structures and classification algorithms are not publicly available and may limit the exact reproducibility of dataset construction.

Case definitions

Heat-associated deaths were defined as deaths in which heat exposure was determined to be a contributing or primary factor based on coroner/medical examiner investigation and SNHD classification. This definition includes both direct heat-related causes, such as heat stroke, and cases in which heat contributed to death in the presence of underlying medical or social risk factors.

Heat-related ED visits were defined using NSSP syndromic surveillance criteria, which identify cases based on combinations of chief complaint text, discharge diagnoses, and clinical indicators consistent with heat-related illness, including heat exhaustion, heat stroke, and dehydration due to heat exposure.

Variables and measures

Variables extracted for analysis included demographic characteristics such as age and sex, as well as social factors, including housing status when available, with particular attention to the identification of individuals experiencing homelessness. Substance use variables were derived from toxicology findings and included the presence of methamphetamine and other substances. Temporal variables included the week of death or ED visit, allowing for the assessment of seasonal trends. Geographic variables included the ZIP code of the incident or residence when available, which were used to evaluate spatial distribution. Environmental data consisted of weekly average temperature measurements for Clark County obtained from publicly available meteorological sources.

Data analysis

Descriptive statistical methods were used to summarize mortality and ED visit data. Continuous variables were reported using means or medians as appropriate, and categorical variables were summarized as frequencies and proportions. Temporal trends were evaluated by examining weekly counts of heat-associated deaths and ED visits in relation to average temperatures. Geographic distribution was assessed by examining the spatial clustering of cases by ZIP code in relation to neighborhood-level socioeconomic characteristics.

No inferential statistical testing or causal modeling was performed, as the primary objective of this study was the descriptive characterization of patterns of heat-associated morbidity and mortality.

Ethical considerations

This study utilized de-identified, aggregate public health surveillance data and did not involve direct interaction with human subjects. The study was reviewed by the University of Nevada, Las Vegas (UNLV) Institutional Review Board and determined to be exempt (UNLV-2026-130).

## Results

Overall mortality and ED utilization

In 2024, Clark County recorded 513 heat-associated deaths (n=513), representing a 73% increase compared to 2023. A total of 3,548 heat-related ED visits (n=3,548) were identified through syndromic surveillance.

The demographic and risk factor profile of individuals experiencing heat-associated mortality is summarized in Table 1.

**Table 1:** Characteristics of heat-associated deaths in Clark County, Nevada, 2024 Summary of demographic and clinical characteristics among individuals with heat-associated mortality, including age distribution, sex, substance involvement, and housing status. Data were derived from the Southern Nevada Health District 2024 Heat-Associated Deaths and Emergency Department Visits Report [27]. Methamphetamine prevalence is reported as a proportion of substance-involved deaths based on Southern Nevada Health District surveillance data. This table was constructed by the authors using aggregated, publicly available data.

Characteristic	Value
Total deaths	513
Male	395 (77%)
Female	118 (23%)
Age 45-64 years	215 (42%)
Age ≥65 years	174 (34%)
Substance involvement	287 (56%)
Methamphetamine (among substance-involved deaths)	60% (n≈172)
Experiencing homelessness	174 (34%)

Demographic characteristics

Heat-associated mortality disproportionately affected males, who accounted for 77% of deaths (n=395), compared to females at 23% (n=118).

Deaths were most common among individuals aged 45-64 years (42%; n=215) and ≥65 years (34%; n=174), indicating a concentration of mortality among middle-aged and older adults.

Substance use and social factors

Substance involvement was identified in 56% of deaths (n=287). Among substance-involved deaths, methamphetamine accounted for 60% (approximately n≈172) and was the most frequently detected substance.

Individuals experiencing homelessness accounted for 34% of deaths (n=174), highlighting a substantial burden among structurally vulnerable populations.

Temporal trends

**Figure 1:**
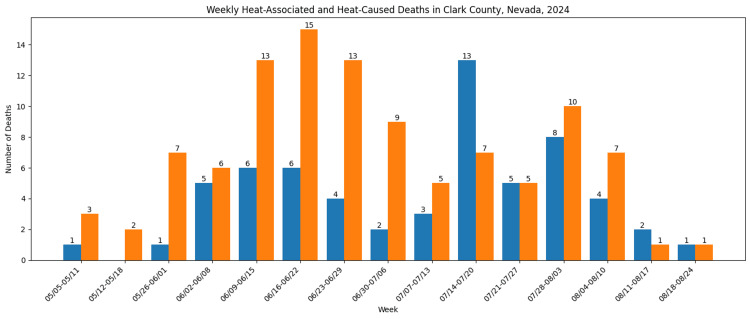
Weekly heat-associated and heat-caused deaths in Clark County, Nevada, 2024 Data obtained from the Southern Nevada Health District 2024 Heat-Associated Deaths and Emergency Department Visits Report [27]. This figure was created by the authors using Microsoft Excel (Microsoft Corporation, Redmond, Washington, United States).

Heat-associated deaths demonstrated a marked seasonal pattern, with the highest mortality occurring during peak summer months. Mortality trends closely paralleled increases in ambient temperature, with the greatest concentration of deaths observed during periods of sustained extreme heat (Figure 1).

**Figure 2:**
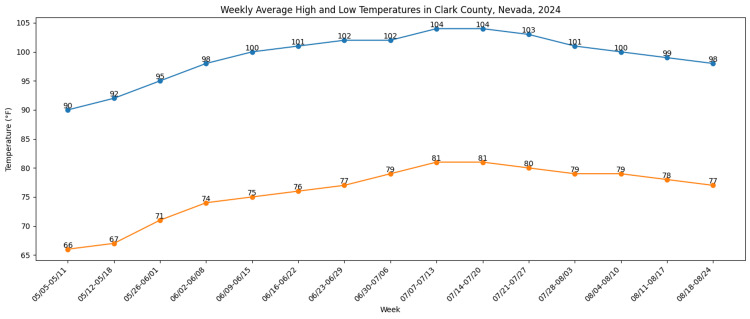
Weekly average high and low temperatures in Clark County, Nevada, 2024 Data obtained from the Southern Nevada Health District 2024 Heat-Associated Deaths and Emergency Department Visits Report [27]. This figure was created by the authors using Microsoft Excel (Microsoft Corporation, Redmond, Washington, United States).

Similarly, ED visits for heat-related illness increased during peak temperature periods, with temporal patterns consistent with mortality trends (Figure 2).

Geographic distribution

**Figure 3:**
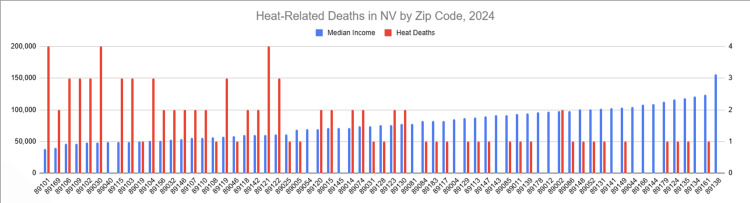
Heat-associated deaths and median household income by ZIP code in Clark County, Nevada, 2024 ZIP codes are arranged in ascending order of median household income. Heat-associated deaths are displayed as ordinal categories (0, 1-4, 5-15, 16-25, >25 deaths) based on publicly reported data. Median household income estimates were obtained from publicly available sources, and mortality data were derived from the Southern Nevada Health District 2024 Heat-Associated Deaths and Emergency Department Visits Report [27]. This figure was created by the authors using Microsoft Excel (Microsoft Corporation, Redmond, Washington, United States).

The geographic distribution of heat-associated deaths by ZIP code demonstrated clustering in specific areas of Clark County (Figure 3). Higher concentrations of deaths were observed in ZIP codes characterized by greater socioeconomic disadvantage and higher proportions of individuals experiencing homelessness.

## Discussion

This study demonstrates a substantial and rising burden of heat-associated mortality and morbidity in Clark County, Nevada, during 2024, with 513 deaths and 3,548 heat-related ED visits. Beyond the magnitude of these findings, the data reveal a consistent and non-random pattern of vulnerability concentrated among specific demographic, behavioral, and socioeconomic groups. Taken together, these results suggest that heat-related mortality in Southern Nevada is not merely an environmental hazard, but a predictable outcome shaped by the interaction of extreme heat exposure and structural vulnerability.

The demographic distribution of deaths aligns with prior epidemiologic research demonstrating increased heat-related mortality among males and older adults [1-3]. However, the substantial proportion of deaths occurring among individuals aged 45-64 years highlights an important and often underrecognized risk among working-age populations. This group may be disproportionately exposed through occupational demands, underlying chronic conditions, or limited access to cooling resources. These findings suggest that prevention strategies should extend beyond traditionally defined elderly populations to include broader at-risk groups.

A central finding of this study is the high prevalence of substance involvement, identified in more than half of heat-associated deaths, with methamphetamine representing the most frequently detected substance. This observation is consistent with existing literature demonstrating that stimulant use impairs thermoregulation, increases metabolic heat production, and diminishes behavioral responses to heat exposure, thereby increasing the risk of hyperthermia and death [12-15]. The co-occurrence of substance use and extreme heat represents a critical and likely underrecognized pathway to mortality that is not adequately addressed in conventional public health approaches focused primarily on environmental exposure.

The disproportionate burden of mortality among individuals experiencing homelessness further underscores the role of social determinants in shaping heat vulnerability. Individuals without stable housing face prolonged environmental exposure, limited access to cooling and hydration, and significant barriers to healthcare access [16,17]. In this context, homelessness should be understood not simply as a demographic characteristic, but as a structural condition that amplifies exposure and limits the capacity to mitigate risk.

The observed geographic clustering of deaths within lower-income ZIP codes reinforces the role of structural and environmental inequities. Prior research has demonstrated that urban heat exposure is unevenly distributed, with higher temperatures occurring in neighborhoods characterized by lower socioeconomic status, reduced vegetation, and historical patterns of disinvestment [20-23]. The spatial patterns identified in this study are consistent with these findings and suggest that built environment factors, including housing quality, access to cooling infrastructure, and neighborhood-level resources, may contribute meaningfully to heat-related risk.

The strong temporal relationship between peak summer temperatures and both mortality and ED utilization reinforces the role of extreme heat as a primary driver of adverse outcomes [28]. The concentration of deaths during periods in which temperatures exceeded 110°F is consistent with prior research demonstrating threshold effects, in which mortality increases sharply once environmental conditions exceed physiologic and adaptive capacity [2,3]. These findings highlight the importance of timely, targeted interventions during periods of sustained extreme heat.

Taken together, these findings support a conceptualization of heat-related mortality as a multifactorial phenomenon driven by the interaction of environmental exposure, behavioral health factors, and structural conditions. This perspective aligns with frameworks of social determinants of health and structural vulnerability, emphasizing that health outcomes are shaped not only by individual characteristics but by access to resources that enable individuals to avoid or mitigate risk. In the context of extreme heat, housing stability, access to cooling, substance use treatment, and community-level infrastructure are central determinants of survival.

Public health and policy implications

These findings have several important implications for public health practice and policy. First, interventions to reduce heat-related mortality should move beyond general population advisories and prioritize targeted strategies for high-risk groups, particularly individuals experiencing homelessness and those with substance use disorders. Expanding access to cooling centers, hydration stations, and mobile outreach services during extreme heat events may reduce exposure among vulnerable populations.

Second, the strong association between substance use and heat-associated mortality highlights an opportunity to integrate heat risk mitigation into behavioral health and substance use treatment programs. This may include patient education, proactive outreach during heat events, and coordination between public health agencies and community-based organizations.

Third, the geographic clustering of deaths underscores the need for place-based interventions. Investments in urban heat mitigation strategies, including increased tree canopy, cooling infrastructure, and housing improvements, may reduce neighborhood-level exposure. Addressing these disparities will require coordination across public health, housing, and urban planning sectors.

Finally, the increasing burden of heat-related mortality highlights the urgency of long-term climate adaptation strategies. As extreme heat events become more frequent and severe, public health systems must develop sustainable approaches to surveillance, early warning, and intervention to prevent avoidable deaths.

Limitations

This study has several limitations. First, the analysis is based on aggregate public health surveillance data, which limits the ability to perform individual-level analyses or adjust for potential confounding variables. As a result, observed associations should be interpreted as descriptive rather than causal.

As an ecological, descriptive analysis using aggregate data, observed associations should not be interpreted as causal, and relationships between individual-level risk factors and outcomes cannot be definitively established.

Second, classification of heat-associated deaths is dependent on coroner and public health determinations and may be subject to misclassification or underreporting, particularly in cases where heat contributes indirectly to mortality.

Third, detailed information on individual-level factors, including comorbid medical conditions, duration and intensity of heat exposure, and behavioral responses, was not available. These factors may influence risk but could not be assessed in this analysis.

Finally, geographic analyses were based on ZIP code-level data, which may not fully capture neighborhood-level variation or individual exposure patterns. Despite these limitations, the study provides a comprehensive descriptive assessment of heat-associated mortality and morbidity in a high-risk urban environment.

## Conclusions

Heat-associated mortality in Clark County, Nevada, represents a substantial and growing public health burden, with marked increases observed in 2024. The findings of this study demonstrate that heat-related deaths are not evenly distributed across the population, but are concentrated among individuals with overlapping vulnerabilities, including substance use, housing instability, and residence in socioeconomically disadvantaged areas. These patterns indicate that heat-related mortality is not solely a function of environmental exposure, but a pattern associated with the interaction between climatic conditions, behavioral health factors, and structural risk.

Reducing heat-related mortality will require a shift from generalized public health messaging to targeted, data-informed interventions focused on high-risk populations. As extreme heat events become more frequent and severe, coordinated strategies that integrate environmental, behavioral health, and social policy approaches will be essential to reducing preventable deaths and improving population health outcomes.
